# Palmitoleic acid protects microglia from palmitate-induced neurotoxicity in vitro

**DOI:** 10.1371/journal.pone.0297031

**Published:** 2024-01-19

**Authors:** Qingting Yu, Yanzhuo Yang, Ting Xu, Yinsheng Cai, Zuisu Yang, Falei Yuan

**Affiliations:** 1 Department of Pharmacy, School of Food and Pharmacy, Zhejiang Ocean University, Zhoushan, China; 2 Zhejiang Puluode Lipid Inc., Zhoushan, China; National Institutes of Health, UNITED STATES

## Abstract

Although palmitoleic acid (POA) is a lipokine with beneficial effects on obesity and is produced as a byproduct from the manufacture of prescription omega-3 fatty acids, its role in nervous system inflammation is still unknown. This study aims to examine the mechanisms and protective effects of POA against palmitic acid (PA)-induced microglial death. PA-induced microglial death was used as a model for POA intervention. Various inhibitors were employed to suppress potential routes of PA entry into the cell. Immunofluorescence staining and Western blotting were conducted to elucidate the protective pathways involved. The results suggest POA has the potential to eliminate PA-induced lactate dehydrogenase (LDH) release, which decreases the overall number of propidium iodide (PI)-positive cells compared with control. Moreover, POA has the potential to significantly increase lipid droplets (LDs) in the cytoplasm, without causing any lysosomal damage. POA inhibited both canonical and non-canonical gasdermin D (GSDMD)-mediated pyroptosis and gasdermin E (GSDME)-mediated pyroptosis, which PA typically induces. Additionally, POA inhibited the endoplasmic reticulum (ER) stress and apoptosis-related proteins induced by PA. Based on the findings, POA can exert a protective effect on microglial death induced by PA via pathways related to pyroptosis, apoptosis, ER stress, and LDs.

## 1. Introduction

Palmitoleic acid (POA), an Omega-7 fatty acid, is a functional fatty acid extracted primarily from marine fish oil and sea buckthorn fruit oil. Marine fish are rich in POA, specifically in the viscera, accounting for almost 10.72% of total lipid contents [1]. While the manufacture of Omega-3 fish oil yields significant quantities of biodiesel, it can also be a good source for extracting POA [2]. Identified as a lipokine in 2008, POA has since received significant attention, with numerous studies focusing on its mechanism of action [3]. Recently, many studies have confirmed that POA plays a key role in the improvement of metabolic syndrome (type 2 diabetes, atherosclerosis, obesity) and cardiovascular diseases [4, 5]. POA’s anti-inflammatory effects have been increasingly studied, with research showing it can alleviate macrophage-mediated inflammation induced by palmitic acid (PA) or lipopolysaccharide (LPS) [6, 7]. Eicosapentaenoic acid (EPA), an Omega-3 fatty acid, is also known for its anti-inflammatory properties [8]. A previous study suggested that a beta-oxidation product of EPA, acetyl-CoA, is recycled into the PA biosynthesis pathway, ultimately resulting in the production of radiolabeled POA [9].

Obesity is closely associated with an enhanced risk for a plethora of diseases, including cardiovascular disease, stroke, type 2 diabetes, and many types of cancers [10, 11]. In recent years, excessive intake of saturated fatty acids has been linked to impaired learning and cognition [12]. Saturated fatty acids such as PA contribute to the onset of metabolic inflammatory diseases, including obesity, in part through hypothalamic dysregulation and degeneration. Saturated free fatty acids PA and lauric acid have both been shown to trigger inflammation in cultured macrophages [13]. As immune cells in the central nervous system, microglia are the first line of defense against external stimuli. PA has been shown to lead to microglial apoptosis and inflammation [14, 15]. In recent years, PA has been found to cause pyroptosis [16], a type of regulated necrosis in which pore-forming protein gasdermin D (GSDMD) executes [17, 18]. In aging human brain tissues and in the brains of patients with Alzheimer’s disease, microglia have been shown to accumulate lipid droplets (LDs), but the role of these LDs-laden microglia has yet to be determined [19]. Macrophages accumulate LDs and become foam cells in the atherosclerotic plaque. PA initiates endoplasmic reticulum (ER) stress and elicits unfolded protein response (UPR). The key mediators in this process are PKR–like ER kinase (PERK) and activating transcription factor 3 (ATF-3) [20]. Astrocytes have recently been found to use saturated fatty acids, including PA, as a tool to activate the p53 up-regulated modulator of apoptosis (PUMA)-mediated apoptotic pathway in neurons and mature oligodendrocytes [21], which involves the release of cytochrome c (Cyt c) from the mitochondria into the cytosol [22].

GPR40 is a G protein-coupled receptor that can be activated by saturated and unsaturated free fatty acids, as well as medium-chain to long-chain fatty acids, such as palmitate, palmitoleate, oleate, and linoleate [23, 24]. This protein is mainly expressed in the brain and pancreas of the human body [23]. Inhibition of GPR40 by DC260126 has been shown to be protective from PA-induced apoptosis in pancreas cells [25]. Additionally, POA has been reported to activate GRP120, another G protein-coupled receptor [26]. Research has shown that the neurotrophin brain-derived neurotrophic factor (BDNF) can significantly increase intracellular levels of POA, while the levels of other known fatty acids remain unchanged [27]. Monounsaturated fatty acids have been found to produce higher lipid accumulation than saturated fatty acids [28]. Fatty acid binding protein 4 (FABP4) has previously been reported to attenuate PA-related inflammation [29]. Cluster of differentiation 36 (CD36) is a membrane protein containing the ability to transport extracellular fatty acids into cells [30]. In this process, a clathrin-coated region on the inner cell membrane forms a vesicle that allows material from a portion of the cell membrane adjacent to it to enter the cell.

The NOD-like receptor protein 3 (NLRP3) inflammasome is mainly found in immune and inflammatory cells. Exposure to stimuli such as LPS has been shown to increase the levels of inactive NLRP3 transcripts. The inflammasome is further activated when NLRP3 oligomerizes and assembles into a complex that includes NLRP3, apoptosis-associated speck-like protein (ASC), and procaspase-1. As a result of this complex, interleukin-1β (IL-1β) and interleukin-18 (IL-18) mature [31].

Although many types of unsaturated fatty acids, including docosahexaenoic acid (DHA) and oleic acid, have been found to play a protective role in PA-induced injury and stress [28], the effect of POA on microglial cell viability is still uncertain.

## 2. Materials and methods

### 2.1 Preparation of fatty acid- bovine serum albumin (BSA) complex

A 50 mM stock solution of sodium palmitate (Aladdin, Shanghai, China) was prepared fresh with distilled water at 70°C. POA (Aladdin, Shanghai, China) was prepared as a 200 mM reserve solution in ethanol. The stock solution of PA and POA was then diluted with preheated (37°C) Dulbecco Modified Eagle’s Medium (DMEM, Procell, Wuhan, China) containing BSA (AS33654, Lanso, Zhejiang, China), with a ratio of PA or POA to BSA of 5:1. Then PA or POA-BSA solution was heated to 37°C while shaking for one hour to allow for complex formation. Subsequently, the PA-BSA and POA-BSA complex solutions were diluted with DMEM and filtered through 0.22 μm pore membrane filters before further use.

### 2.2 Cell viability and lactate dehydrogenase (LDH) assay

BV-2 cells were obtained from Hunan Fenghui Biotech Inc. (CL0056) and cultured at 37°C and 5% CO_2_ in a humidified incubator containing 10% fetal bovine serum (FBS, Biological Industries Ltd., Israel). Prior to treatment, the complete medium was substituted with DMEM without FBS.

BV-2 cells were seeded onto plates (2×10^4^ cells/well for 96-well plate). Before treatment, medium was replaced by FBS-free medium. Inhibitors (sulfosuccinimidyl oleate, SSO, Cayman; DC260126, MedChemExpress; AH7614, ApexBio; HTS01037, HTS, Cayman; Pitstop 2, MedChemExpress) were added one hour prior to PA treatment. Then PA, POA, or vehicle (BSA) was added to the medium. After treatment, cells were incubated for two hours with 10% cell counting kit-8 (CCK-8, K1018, ApexBio, Houston, TX). The absorbance at 450 nm was measured via microplate reader (SpectraMax M2, Molecular Devices, CA). Cell viability is expressed as a percentage of control.

BV-2 cells were seeded on 96-well plates and treated with BSA, PA, or PA plus POA for four hours or 24 h. The supernatant post-treatment was tested with the LDH Cytotoxicity Assay Kit (C0017, Beyotime, Shanghai, China) according to manufacturer’s instructions.

### 2.3 Immunofluorescence, boron-dipyrromethene (BODIPY) staining, and terminal deoxynucleotidyl transferase dUTP nick end labeling (TUNEL) staining

After treatment, BV-2 cells were fixed with 4% paraformaldehyde for 20 min then permeated by 0.3% Triton X-100 for 10 min and subsequently blocked by 1% BSA for 20 min. Primary antibodies (galectin-3, ab76245, Abcam, 1:400; lysosomal associated membrane protein 1, LAMP1, ab208943, Abcam, 1:400; Lipid A, GTX40001, GeneTex, 1:400; cytosolic phospholipase A2-α, cPLA2, sc-454, Santa Cruz, 1:100; Cyt c, 66264–1, Thermo Fisher, 1:400) were incubated with cells overnight at 4°C. Fluorescence conjugated secondary antibodies (Jackson ImmunoResearch, 711-025-152, 705-095-147, 115-095-003, 715-585-150, 1:200) were added at room temperature for one hour.

After fixing with 4% paraformaldehyde, cells were stained with BODIPY™ 493/503 (Invitrogen, Life Technologies, Carlsbad, CA, USA) or PI (Beyotime, Shanghai, China) for 20 min at room temperature. TUNEL staining (MK1027, BOSTER, Wuhan, China) was performed following the manufacturer’s instructions.

Cell nuclei were stained with 4’,6-diamidino-2-phenylindole (DAPI), and all images were captured with fluorescent microscopy (Olympus BX41).

### 2.4 Western blotting

Protein was extracted with radio immuno precipitation assay (RIPA) buffer (Solarbio, Beijing, China) separated and transferred to polyvinylidene difluoride (PVDF) membranes. Membranes were blocked with 5% BSA for one hour and then incubated with the primary antibodies (cleaved caspase-1, 89332, CST; cleaved caspase-3, 9661T, CST; GSDMD-N, NBP2-33422, Novus; caspase-11, NB120-10454, Novus; GSDME, ab215191, Abcam; poly (ADP-ribose) polymerase, PARP, 9532, CST; cleaved IL-1β, 63124, CST; ASC, 67824, CST; NLRP3, DF7438, Affinity; PUMA, 55120–1, Proteintech; ATF-3, SC-81189, Santa Cruz; PERK, SC-377400, Santa Cruz; α-Tubulin, T9026, Millipore; all the above antibodies were diluted as 1:1000) at 4°C overnight. Membranes were incubated with horseradish peroxidase conjugated secondary antibodies (S0009, Affinity; 111-035-003 & 115-035-003, Jackson ImmunoResearch) at room temperature for one hour. Finally, the protein bands were detected with ECL Western Blotting Substrate (Solarbio, Beijing, China) and quantified using Alphaview SA software for Fluor Chem FC3 (ProteinSimple, San Jose, CA, USA). All Western blots were performed at least three replicates.

### 2.5 ELISA

Cell culture medium was centrifuged, collected, and analyzed according to manufacturer’s instructions for both QuantiCyto® Mouse IL-1β ELISA Kit (NeoBioscience, Shenzhen, China) and Mouse IL-18 ELISA Kit (Elabscience, Wuhan, China).

### 2.6 Statistical analysis

Experiments in this study were performed in independent triplicate. Statistical analysis was performed using GraphPad Prism 9.0 (GraphPad Software Inc., San Diego, CA, USA). Data are shown as mean ± standard deviation (SD). Significance analysis using one-way analyses of variance (ANOVA) followed by Tukey’s multiple comparisons test or unpaired t test. *P* < 0.05 were considered statistically significant.

## 3. Results

### 3.1 POA protects microglia from PA injury

**Fig 1:**
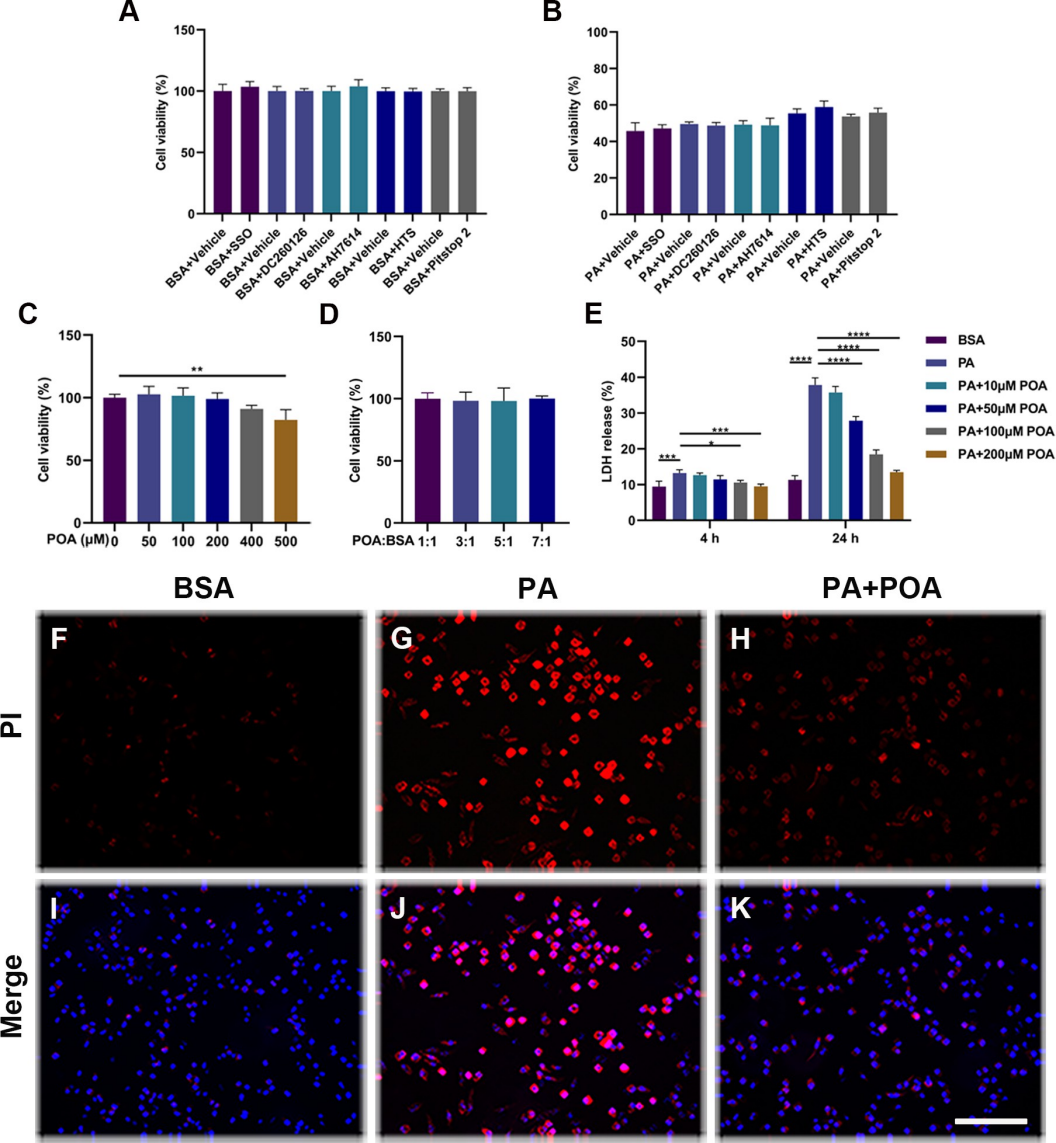
POA protects from PA injury. (A) BV-2 cells were pretreated with inhibitors or vehicles for one hour, followed by treatment with BSA for 24 h for cell viability assay. (B) BV-2 cells were pretreated with inhibitors or vehicles for one hour, followed by treatment with PA for 24 h for cell viability assay. (C) Effect of POA on BV-2 cell viability at different concentrations. (D) Effect of different POA:BSA ratios on BV-2 cell viability. (E) PA induced effect of POA on LDH release from BV-2 cells. (F-H) PI staining (red) of cells treated with BSA, 200 μM PA, 200 μM PA+200 μM POA for four hours. (I-K) PI staining merged with DAPI (blue). Data are shown as mean ± SD. *p < 0.05, **p < 0.01, ***p < 0.001, ****p < 0.0001. Scale bar: 100 μm.

The mechanism through which PA enters the cell has yet to be deduced, so we decided to investigate whether the injury of BV-2 cells induced by PA was related to its transport into cells. To do this, we pretreated cells with various inhibitors such as SSO, DC260126, AH-7614, HTS, and Pitstop 2 for one hour to inhibit CD36, GPR40, GPR120, FABP4, and clathrin, respectively. We found that inhibition of these proteins had no significant effect on microglial death (*p* > 0.05) (Fig 1A and 1B), suggesting that cell death from PA injury may be an upstream event or that these proteins do not have a direct effect on PA entrance to the cell. Furthermore, we investigated whether POA has the potential to reduce microglia cell death induced by PA. To determine the effect of POA on BV-2 cells, cell viability after 24 h of treatment with different concentrations of POA was analyzed via CCK-8 method. As shown in Fig 1C, when the concentration of POA was under 400 μM, no significant effect on BV-2 cell viability was seen (*p* > 0.05). However, when the concentration of POA reached 500 μM, the viability of BV-2 cells was reduced by 17.7% compared with the control group (*p* < 0.01). To investigate whether the ratio of POA:BSA has any effect on the cell viability of BV-2 cells [32], we evaluated the effect of the differing ratios of POA:BSA (1:1, 3:1, 5:1, 7:1) on the viability of BV-2 cells. Our results suggest that these ratios had no significant effect on the cell viability of BV-2 cells (*p* > 0.05, Fig 1D). Thus, POA concentrations below 200 μM were selected to evaluate their effect on injury caused by 200 μM PA in BV-2 cells. Compared to the control groups, after four hours and 24 h of 200 μM PA treatment, LDH release increased by 40.3% (*p* = 0.0002) and 234.0% (*p* < 0.0001) respectively. PA treated BV-2 cells were treated with 10μM, 50μM, 100μM, and 200 μM of POA for four hours, respectively. Compared to the group treated with PA alone, LDH release was decreased by 5.7% (*p* = 0.9607), 18.5% (*p* = 0.1380), 27.6% (*p* = 0.0109), and 38.9% (*p* = 0.0004), respectively. 24 h post-treatment, LDH release was found to decrease by 18.5% (*p* = 0.3187), 87.7% (*p* < 0.0001), 170.9% (*p* < 0.0001), and 214.6% (*p* < 0.0001), indicating the protective effect of POA is dose-dependent (Fig 1E). There was no significant difference between the control group and the PA+200 μM POA group (*p* > 0.05), suggesting that the protection brought by POA is highly effective (Fig 1E). These results were further confirmed via PI staining. PI staining of cells treated with BSA, PA, and PA plus 200 μM POA treated cells for four hours, showed that POA led to a significant decrease in the number of PI positive cells treated with PA (Fig 1F–1K).

### 3.2 The protection of POA is via ER stress and apoptotic pathways

**Fig 2:**
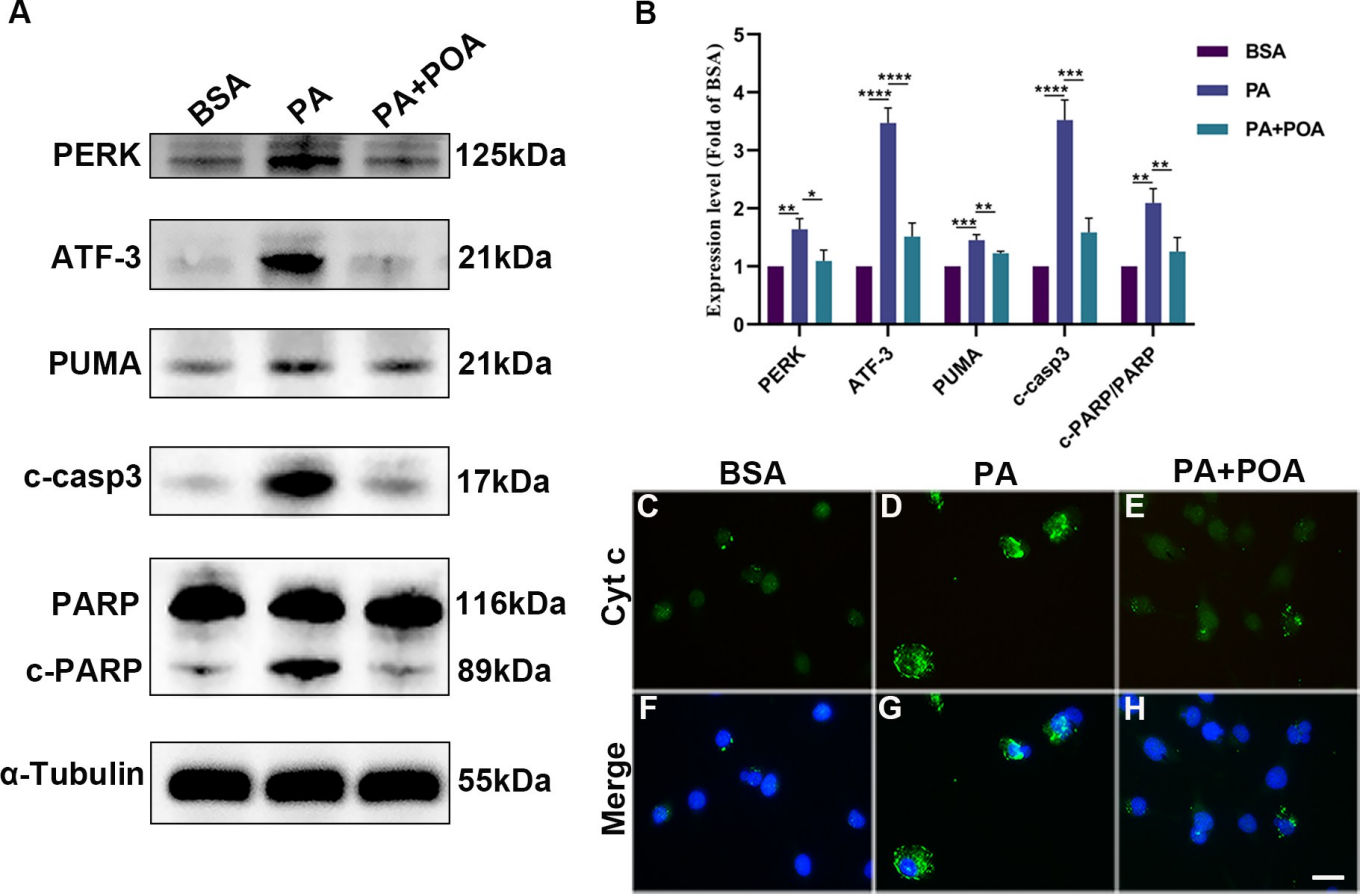
POA protects against PA-induced cell death via the ER stress and apoptotic pathways. (A, B) Cells were treated with BSA, 200 μM PA, 200 μM PA+200 μM POA for four hours, and protein levels of PERK, ATF-3, PUMA, cleaved caspase-3 (c-casp3), PARP, cleaved PARP (c-PARP) were detected via Western blot. (C-H) Immunofluorescence staining of Cyt c (green) merged with DAPI (blue). Data are shown as mean ± SD. *p < 0.05, **p < 0.01, ***p < 0.001, ****p < 0.0001. Scale bar: 20 μm.

To further determine the protective mechanism of POA against microglial death induced by PA, we analyzed the expressions of proteins associated with ER stress and apoptosis. As shown in Fig 2A, Western blots showed PA treatment significantly increased the expression levels of ER stress and apoptosis-related proteins. The levels of PERK, ATF-3, PUMA, cleaved caspase-3, and cleaved PARP increased by 64% (*p* = 0.0049), 247% (*p* < 0.0001), 55% (*p* = 0.0002), 252% (*p* < 0.0001), and 110% (*p* = 0.0013) in comparison to the BSA treated group. The levels of PERK, ATF-3, PUMA, cleaved-caspase3, and cleaved-PARP were reduced by 33% (*p* = 0.0107), 56% (*p* < 0.0001), 20% (*p* = 0.0066), 55% (*p* = 0.0002), and 40% (*p* = 0.005) in the PA+POA group compared to the PA group, respectively (Fig 2A and 2B). As shown in Fig 2C–2H, Cyt c was diffusely distributed in the cytoplasm of PA-treated microglia, but it was evenly distributed in the BSA and PA+POA groups.

### 3.3 POA also functions through inhibiting pyroptosis

**Fig 3:**
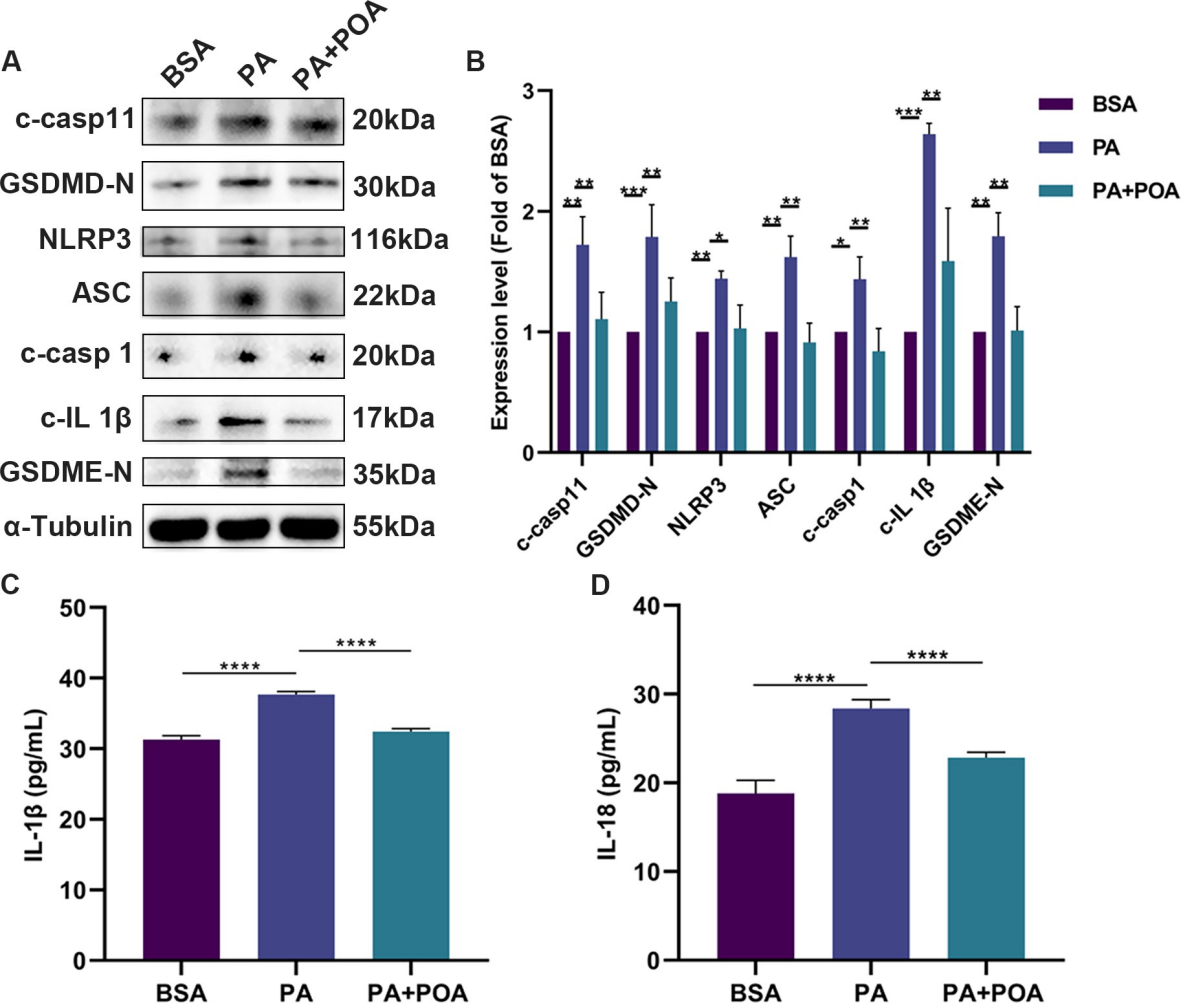
POA protects PA-induced cell death via pyroptotic pathways. (A, B) Cells were treated with BSA, 200 μM PA, 200 μM PA+200 μM POA for four hours, and protein levels of cleaved caspase-11 (c-casp11), GSDMD-N, NLRP3, ASC, cleaved caspase-1 (c-casp1), cleaved IL-1β (c-IL 1β), GSDME-N were subsequently detected by Western blot. (C, D) Concentrations of IL-1β and IL-18 in cell culture supernatants were determined via ELISA. Data are shown as mean ± SD. *p < 0.05, **p < 0.01, ***p < 0.001, ****p < 0.0001.

To determine whether the protective effect of POA against microglia death caused by PA was related to pyroptosis, proteins for canonical and non-canonical pyroptosis pathways were analyzed. The protein levels of cleaved caspase-11, GSDMD-N, NLRP3, ASC, cleaved caspase-1, and cleaved IL-1β increased by 72% (*p* = 0.0010), 79% (*p* = 0.0007), 44% (*p* = 0.0091), 62% (*p* = 0.0036), 44% (*p* = 0.0299), and 164% (*p* = 0.0006) in the PA treated group compared to the BSA treated group. The levels of cleaved caspase-11, GSDMD-N, NLRP3, ASC, cleaved caspase-1, and cleaved IL-1β were reduced by 36% (*p* = 0.003), 30% (*p* = 0.0083), 29% (*p* = 0.0124), 44% (*p* = 0.0018), 42% (*p* = 0.0073), and 40% (*p* = 0.0059), respectively, in the PA+POA treated group compared to the PA treated group (Fig 3A and 3B). In addition, the expression of GSDME-N was increased by 79% after PA treatment (*p* = 0.0023) and decreased by 44% in the PA+POA group (*p* = 0.0025). Furthermore, we examined the levels of IL-1β and IL-18 in the cell supernatant via ELISA. We found that the release of IL-1β and IL-18 increased by 20% (*p* < 0.0001) and 80% (*p* = 0.0018), respectively, after PA treatment compared to BSA treatment, and decreased by 14% (*p* < 0.0001) and 28% (*p* = 0.025) after POA treatment, compared to the PA group (Fig 3C and 3D).

### 3.4 PA causes lysosomal leakage and POA-induced LDs may be the protective mechanism

**Fig 4:**
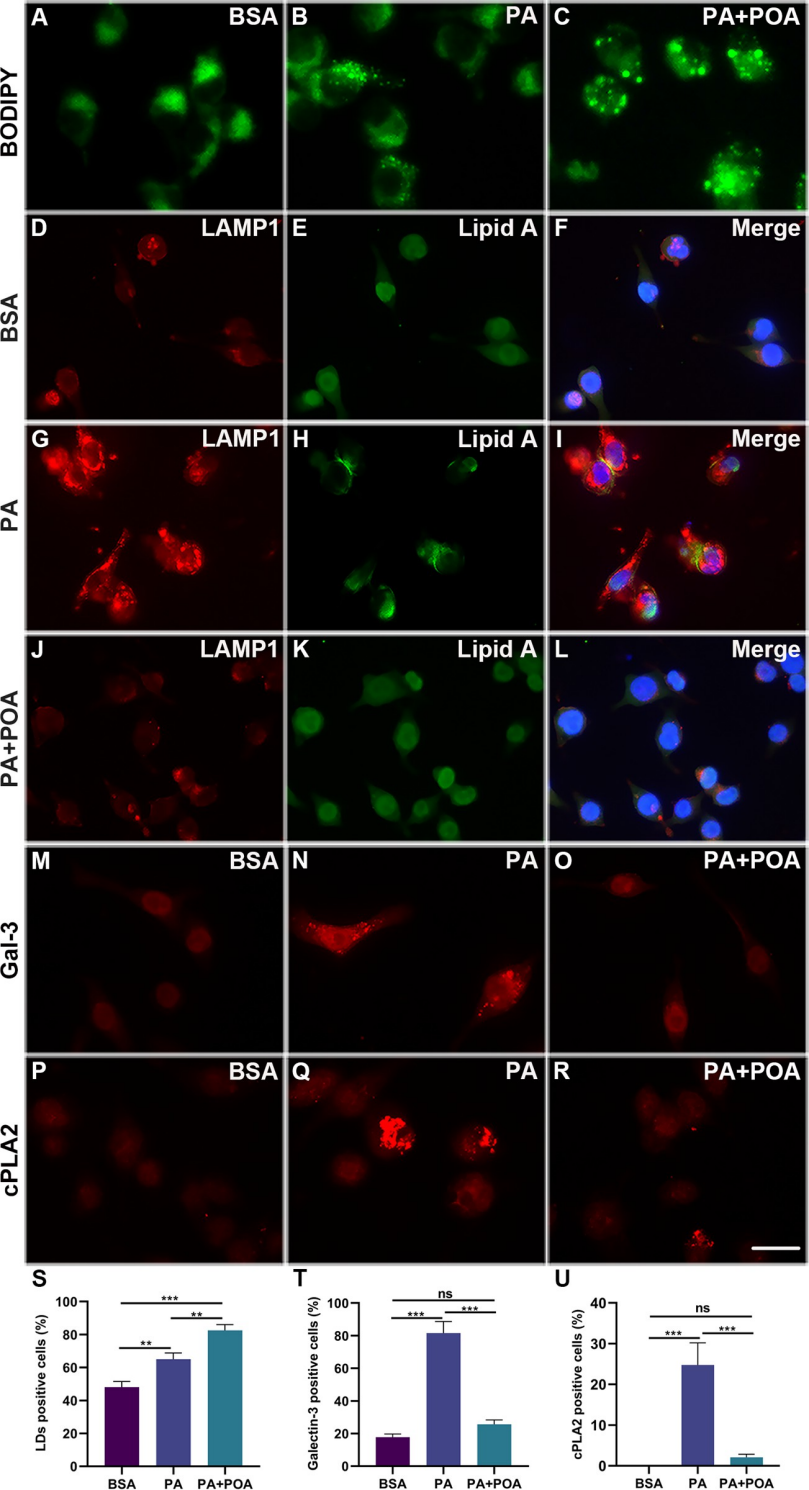
POA-induced LDs may be the protective mechanism. (A-C) Representative images of BODIPY staining. (D-L) Cells were treated with BSA, 200 μM PA, 200 μM PA+200 μM POA for four hours, and immunofluorescence staining was performed for LAMP1 (red), Lipid A (green) and merged with DAPI (blue). (M-O) Representative images of galectin-3 (Gal-3) immunofluorescence staining. (P-R) Representative images of cPLA2 immunofluorescence staining. (S-U) The percentage of LDs, Gal-3 and cPLA2 in the different group. Data are shown as mean ± SD. **p < 0.01, ***p < 0.001. Scale bar: 20 μm.

To determine the effects of PA and PA+POA on LDs in cells, cells were treated with BSA, 200 μM PA, and 200 μM PA plus 200 μM POA for four hours. Cells were then fluorescently labeled with BODIPY™ 493/503 for LDs. Our results show that the LDs in the BSA treated group were sparse and diffusely distributed, with an overall cell count loaded with LDs at 48.1% ± 3.4% (Fig 4A and 4S). Conversely, the overall LDs count increased to 65.1% ± 3.8% in the PA treated group (Fig 4B and 4S, *p* = 0.0029). Surprisingly, the PA+POA exhibited a significant increase in LDs, with an overall LDs count of 82.5% ± 3.6%, accompanied by aggregation (Fig 4C and 4S, vs. the BSA treated group, *p* < 0.001; vs. the PA treated group, *p* = 0.0025). We further discovered intact lysosomes (LAMP1+) in both the BSA treated group and the PA+POA treated group (Fig 4D and 4J). However, in PA treated cells, the lysosomes were found to be damaged (Fig 4G). Similarly, LPS (Lipid A) was uniformly distributed around the nucleus in both BSA and PA+POA treated groups (Fig 4E, 4F, 4K and 4L); however, it was distributed throughout the cytosol after PA treatment (Fig 4H and 4I). To further confirm that lysosomes were ruptured after PA treatment, immunofluorescence staining of galectin-3 was performed, as galectin-3 has been shown to mediate repair and removal of damaged lysosomes [33]. The percentage of galectin-3 positive cells was 17.7% ± 2.0%, 81.6% ± 7.1%, and 25.7% ± 2.6% in the BSA treated group, the PA treated group and PA+POA treated group, respectively (Fig 4T). We discovered a significant quantity of galectin-3 positive cells in the PA treated group (vs. the BSA treated group or PA+POA treated group, *p* < 0.001, Fig 4N); however, both the BSA treated and PA+POA treated groups contained very few galectin-3 positive cells (the BSA treated group vs. the PA+POA treated group, *p* = 0.1611, Fig 4M and 4O). Since cPLA_2_-α has been shown to co-localize with LDs induced by LPS [34], we conducted our own study for confirmation and discovered that cPLA_2_-α was activated in the PA treated group (24.8% ± 5.5% positive cells, vs. the BSA treated group or PA+POA treated group, *p* < 0.001). In contrast, the PA+POA treated group contained only 2.1% ± 0.7% cPLA2-α positive cells, which were not significantly different from the BSA treated group (*p* = 0.7089, Fig 4P–4R and 4U). Therefore, this suggests LDs formation in the PA group may come from leaking LPS, while the LDs in the PA+POA treated group do not.

## 4. Discussion

**Fig 5:**
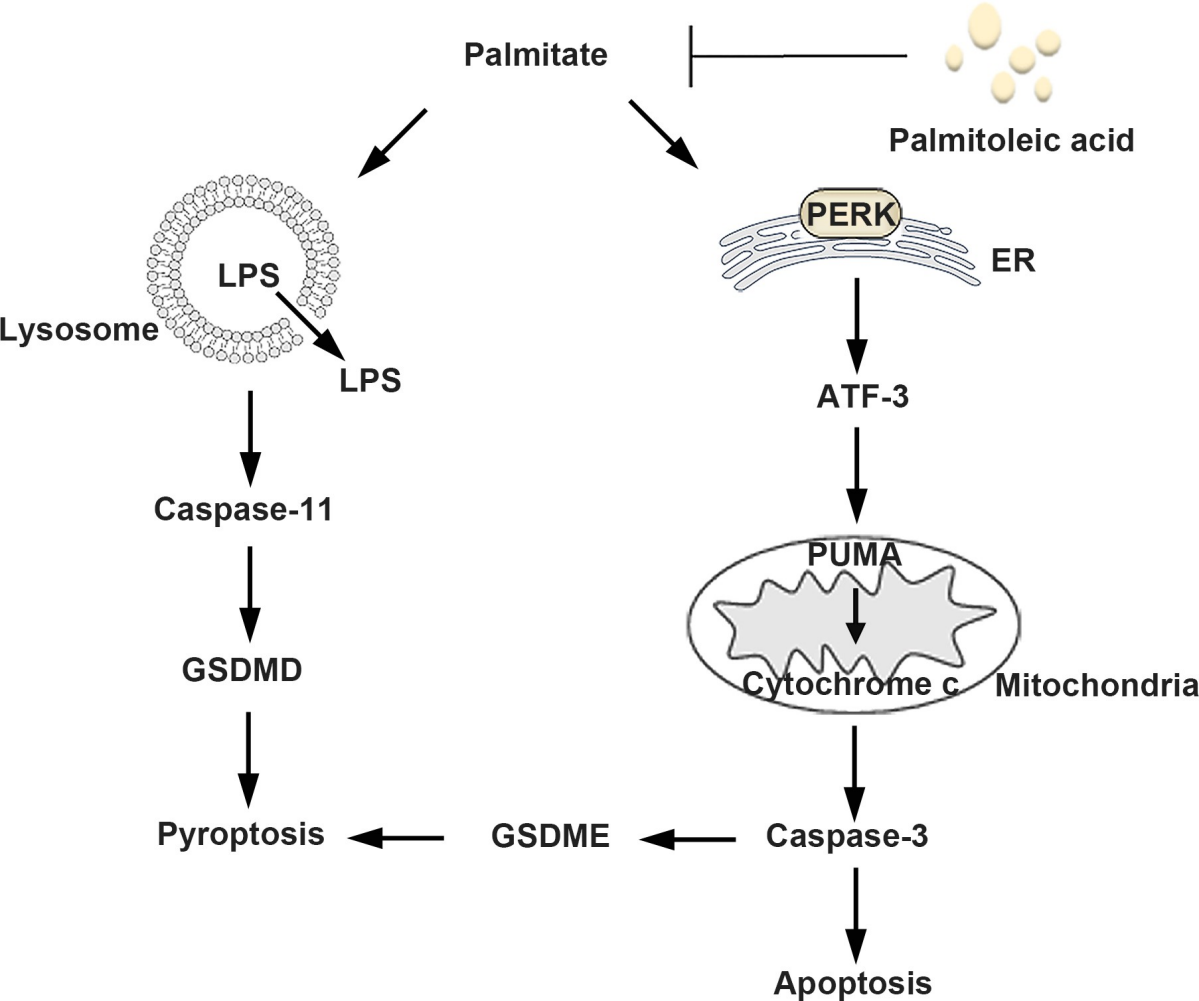
Mechanistic model of the protective effect of POA on PA-induced microglia death.

In the present study, we investigated the protective function of POA in microglia by co-administering PA and POA. Our results showed that POA effectively attenuated PA-induced injury by inhibiting pyroptosis and apoptosis. It was observed that the protective effect of POA was dependent on its concentration. Our data suggest that POA’s protective effect is an upstream event, which may be associated with the formation of LDs. The mechanistic model of POA’s protective effect is illustrated in Fig 5.

Despite the widespread availability of sea buckthorn in the northwest region of China, it is primarily utilized for soil and water conservation rather than commercial purposes. Consequently, the yield of sea buckthorn fruit oil is low, expensive, and insufficient to meet the demand for POA in various industries, including health supplementation. Commercial POA is available as a byproduct of the Omega-3 supplement industry, mainly derived from the light fractions of molecular distillation that is usually used as biodiesel. Marine fisheries offer an abundance of resources, including fish oil that can be sourced from various species. This makes marine fish oil an important and potentially lucrative source of POA. Additionally, the high concentration of POA in marine fish oil facilitates animal studies to further explore its physiological functions [35]. This is particularly significant because fatty acids, including POA, are known to be capable of crossing the blood-brain barrier (BBB), making further studies on the mechanisms underlying POA’s effects on the central nervous system possible [36].

Our results indicate that PA injury was not mediated through GPR40 and GPR120, consistent with the findings reported results produced by Urso et al [28]. Despite their study proposing that the inhibition of CD36 rescued PA-induced microglia cell death using the same inhibitors and treatments as our study, our results suggest that PA toxicity in our study was not linked to PA uptake mediated by CD36. In prior research, oleic acid, another common monounsaturated fatty acid, has been shown to protect against PA-induced hepatocyte death by inhibiting ER stress and pyroptosis [16]. This parallels our microglia model closely.

The beneficial effects of POA on LPS-induced inflammation might also be associated with LDs formation as LPS toxicity exists in the form of lipid. Interestingly, LPS treatment alone had also been found to cause LDs formation [34]. Furthermore, LDs have been suggested to protect against various types of cellular stress, including lipotoxic stress [37]. Therefore, the formation of LDs by LPS may be a manifestation of the body’s defense response. LDs were wrapped by a membrane protein called perilipin [38]. It would also be prudent to investigate whether LDs formation is involved in POA lipoprotection via perilipin knockdown. Galectin-3 expression has also been shown to be significantly upregulated in PA-induced macrophages, consistent with the results of this study [39]. The aggregation of galectin-3 further suggests that PA leads to the rupture of lysosomes.

NLRP3 has been found to sense PA stimulation, and it has been reported that only 3 days of high-fat diet (HFD) feeding can increase the gene expression of NLRP3 in microglia [40]. However, it is not further explained whether the downstream of the NLRP3 inflammasome is related to pyroptosis [41]. Our work suggests that NLRP3 inflammasome activation is associated with the upregulation of pyroptosis-related proteins. GSDMD can also be cleaved indirectly through caspase-11 mediated activation of the NLRP3 inflammasome and subsequent caspase-1 cleavage [42]. Whether NLRP3 inflammasome is activated indirectly by caspase-11 or directly by PA in our work awaits further study.

GSDME is expressed in our cell model, and plays a significant role in mediating pyroptosis [43]. Although apoptotic cell death is confirmed by PARP-1 and TUNEL staining (Fig 2 and S1 Fig), the elevated level of caspase-3 suggests GSDME-mediated pyroptosis is probable. Our study found that both apoptosis and pyroptosis were present in PA-induced microglial cell death. Furthermore, we found that pyroptosis is not only mediated by GSDMD, but also by GSDME. Caspase-3 was found to mediate not only apoptosis, but also cleavage of GSDME leading to pyroptosis [44]. Apoptosis and GSDMD/GSDME-mediated pyroptosis are associated with each other and involve similar proteins. It has been reported that the mitochondrial permeability transition (MPT) activates caspase-4 (counterpart of caspase-11 in humans) by promoting the assembly of a protein complex (apoptosis protease activating factor 1, Apaf-1/pyrosome), which further cleaves caspase-3 to mediate GSDME-induced pyroptosis [32]. It has been suggested that caspase-1 is also able to promote the cleavage of caspase-3/7 and promote apoptosis. Further cleavage of amino acid residue site Asp87 of GSDMD during apoptosis has been shown to inhibit the pyroptotic activity of GSDMD. This expands our understanding on the complex interplay between these cell death pathways [45].

This study is the first to find that POA alleviates PA-induced damage to microglia by inhibiting pyroptosis, apoptosis and ER stress pathways. The formation of POA-induced LDs may be a key event in protecting microglia from death. The concentration of POA used in this study falls within the physiological range in human serum and should therefore be considered safe for in vivo treatment [46]. A previous study has demonstrated that HFD-induced inflammation and reactive microgliosis occur in the hypothalamus of rodents [47]. The physiological significance of this microgliosis and the impact of POA consumption on brain function are still unknown. Nevertheless, POA has been shown to significantly inhibit liver inflammation induced by HFD [48]. The current study has its limitations: in this cell culture model, POA has a direct effect on the cell. For in vivo studies, we must first address the challenge of the bioavailability of POA and how it enters the BBB. Since DHA, PA, and oleic acid can cross the BBB through the transporter Mfsd2a in the form of lysophosphatidylcholine, it will be of interest to determine whether POA employs the same strategy to traverse the BBB [49]. BV-2 microglia are an immortalized cell line, and primary culture from the mouse brain should be conducted before animal studies.

In the future, we intend to determine what cell types have the potential to be protected by POA, and whether the brain is able to use the formation of LDs as a strategy to combat obesity and inflammation.

## Supporting information

S1 Fig POA protects against PA-induced apoptosis. (PDF)

S1 Raw images Original images for blots and gels. (PDF)

S1 Data Raw data of this article. (XLSX)
