# Supplementary material for: Palmitoleic acid protects microglia from palmitate-induced neurotoxicity in vitro
Source: PLoS One. 2024 Jan 19;19(1):e0297031. doi: 10.1371/journal.pone.0297031 (PMC10798504; doi:10.1371/journal.pone.0297031)
Supplement: S1 Fig — (PDF) [file pone.0297031.s001.pdf]

Supplementary Figures

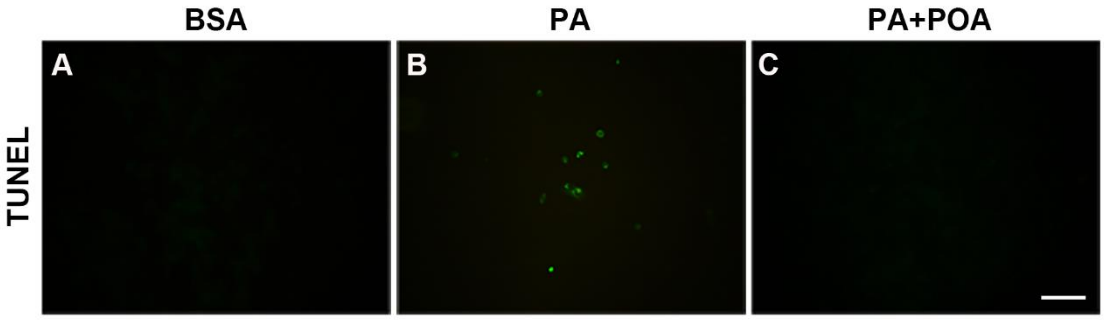

S1 Fig. POA protects against PA-induced apoptosis. (A-C) BV-2 cells were treated with BSA, 200 μM PA, 200 μM PA+200 μM POA for 24 h and then detected by TUNEL staining. Scale bar: 100 μm.
