# Supplementary figures and images for: Palmitoleic acid protects microglia from palmitate-induced neurotoxicity in vitro
Source: PLoS One. 2024 Jan 19;19(1):e0297031. doi: 10.1371/journal.pone.0297031 (PMC10798504; doi:10.1371/journal.pone.0297031)

Original images for blots and gels

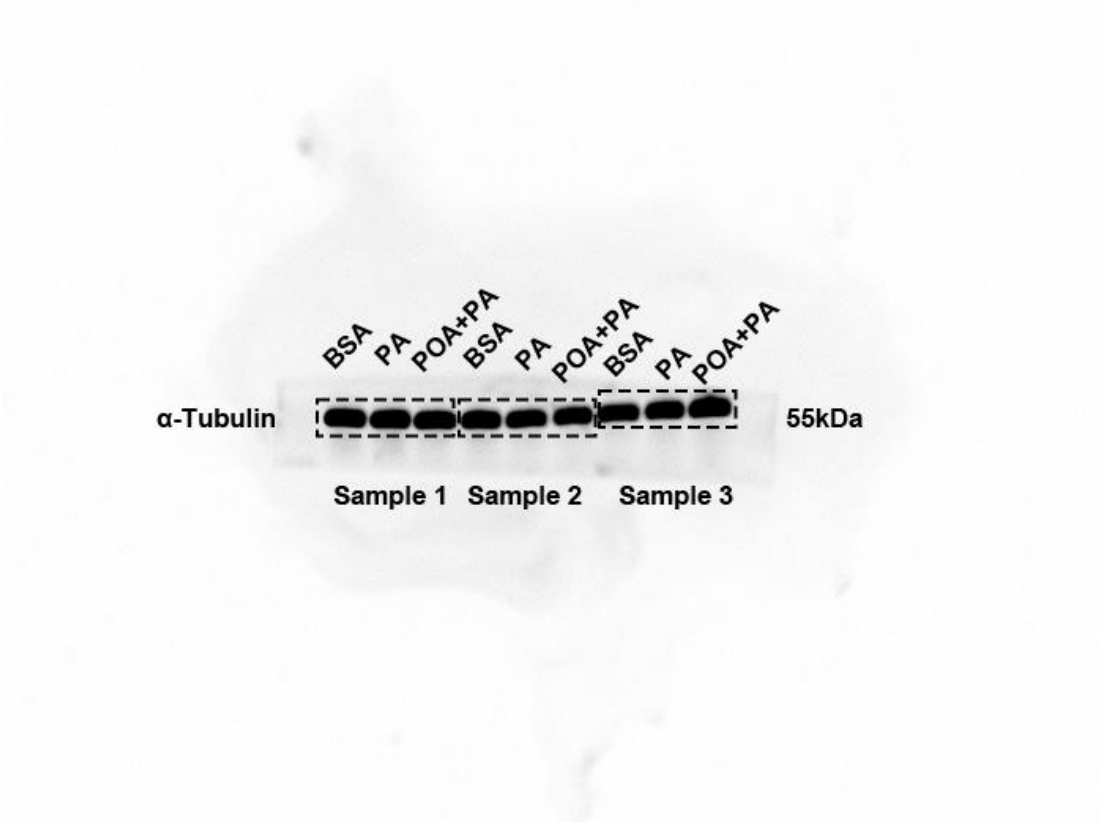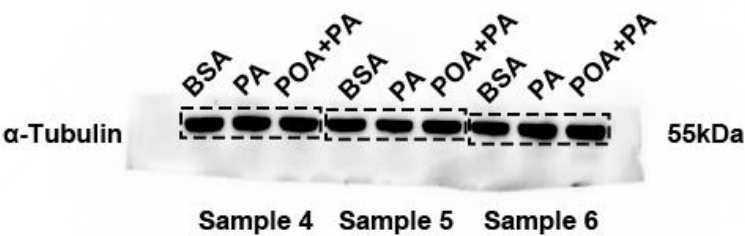

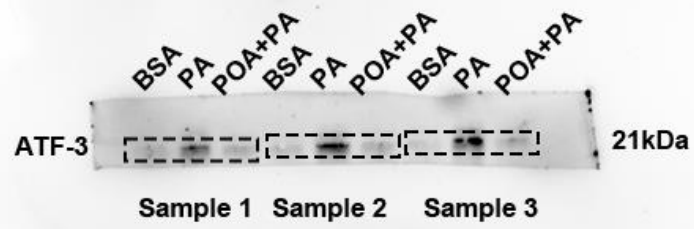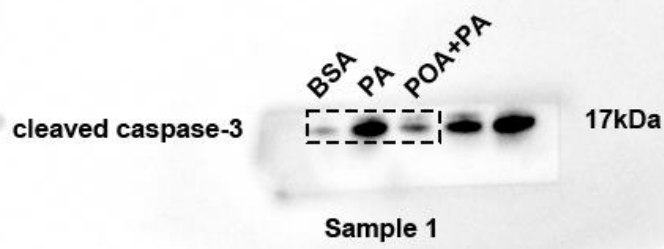

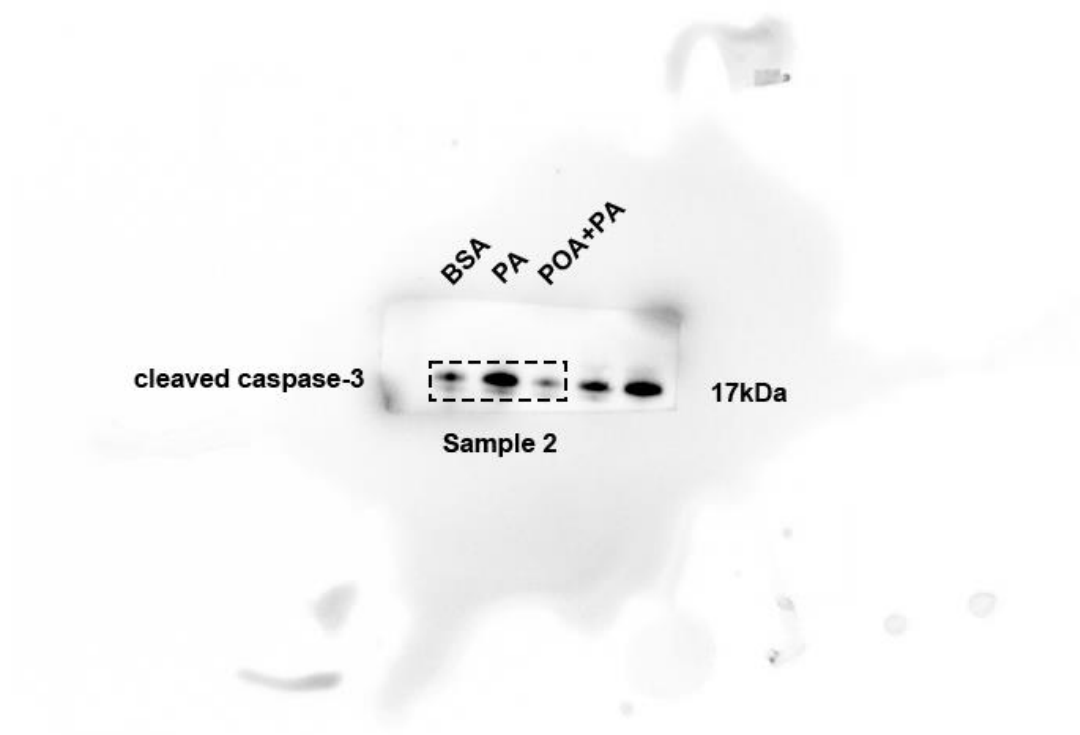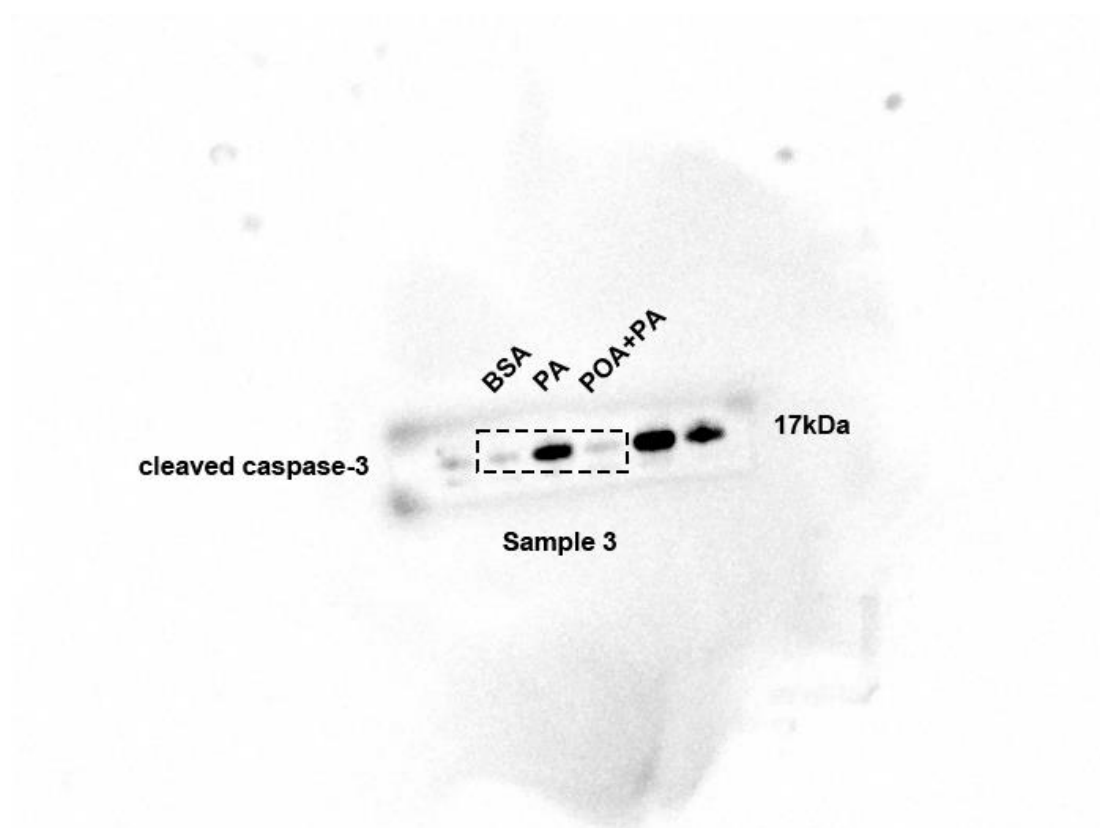

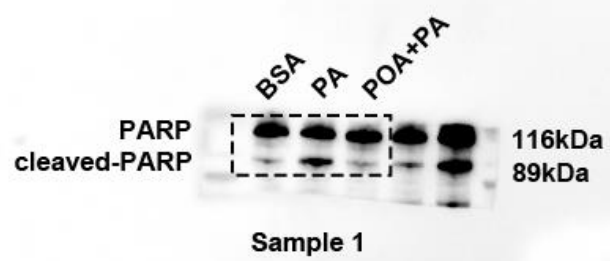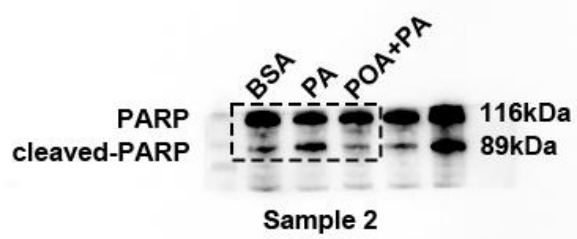

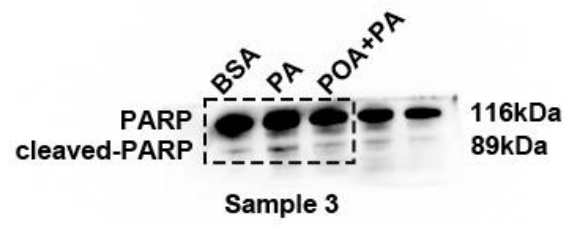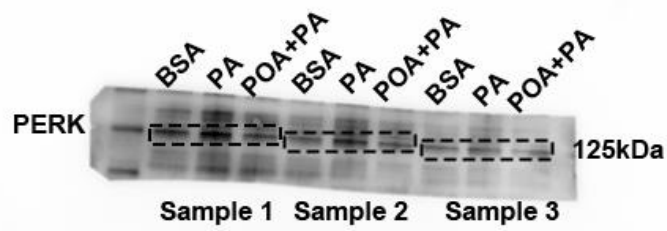

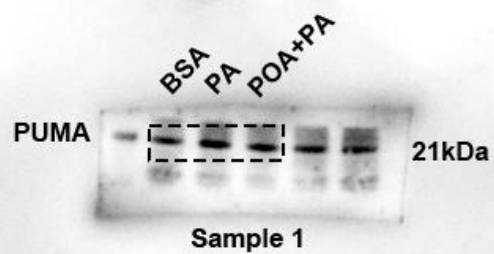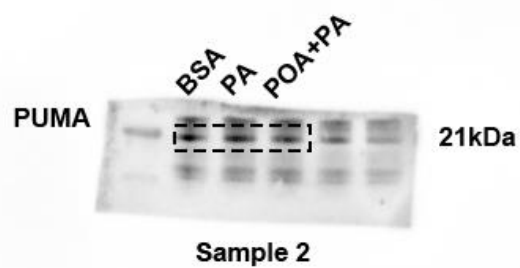

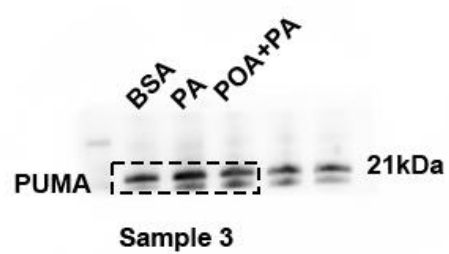

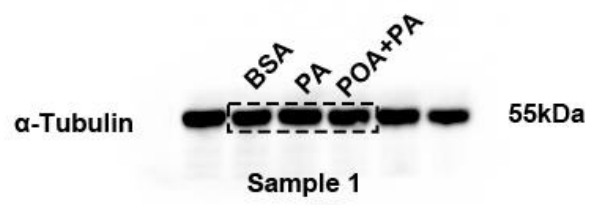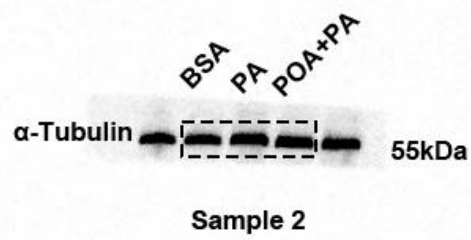

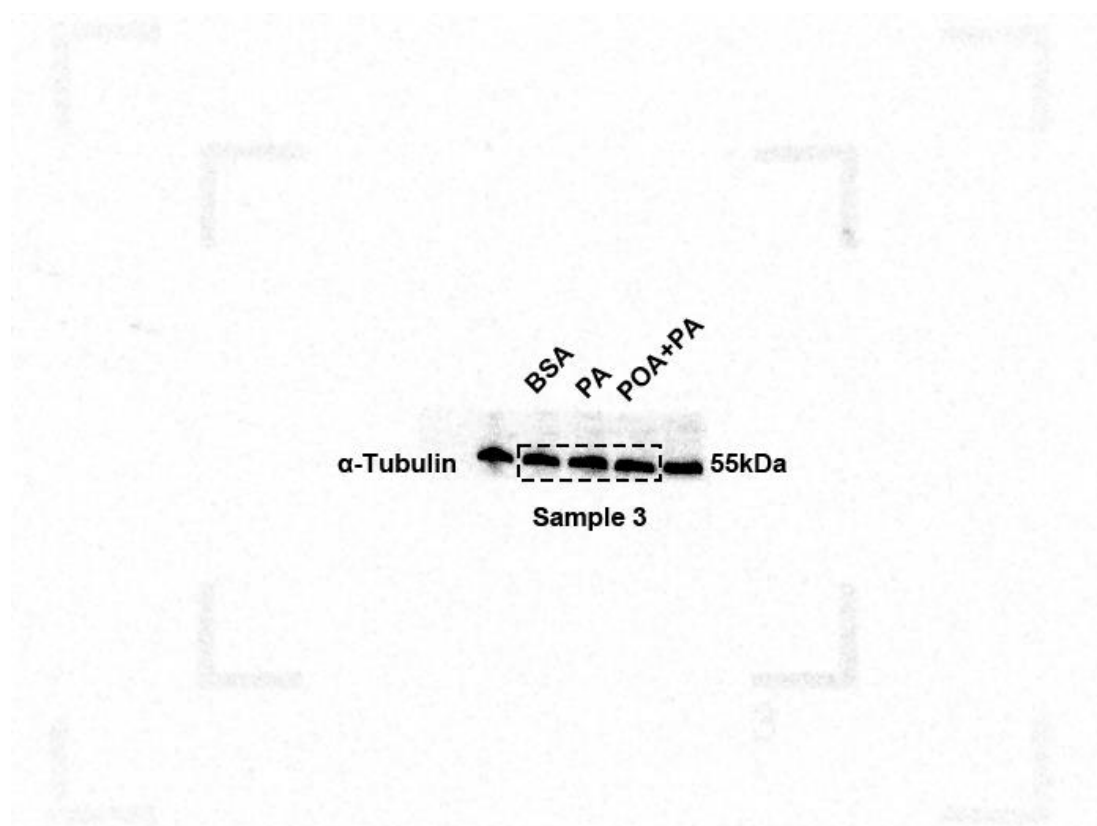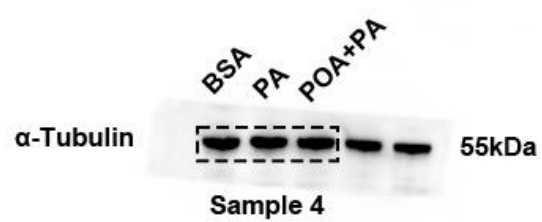

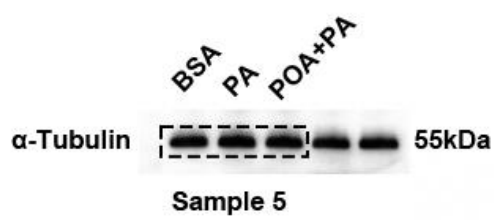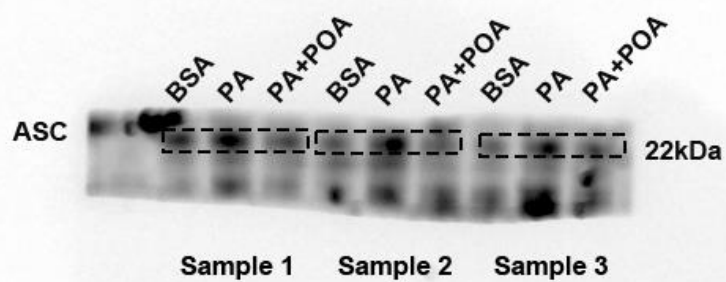

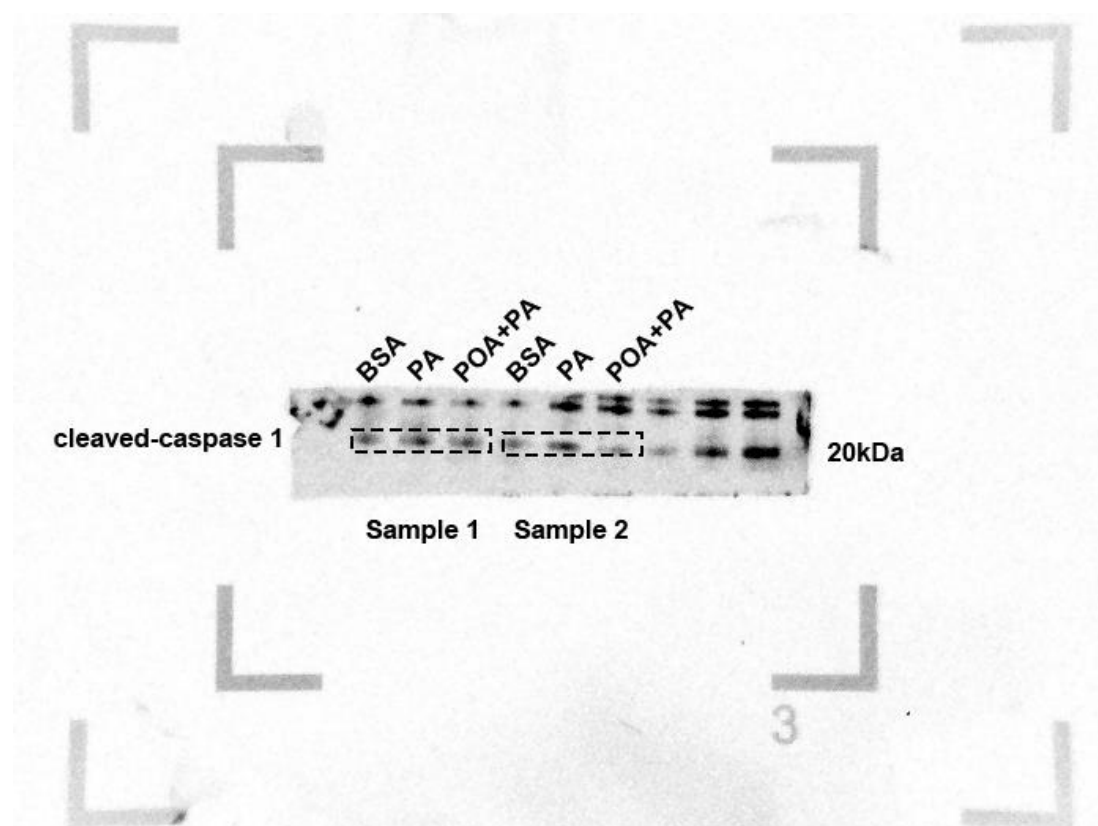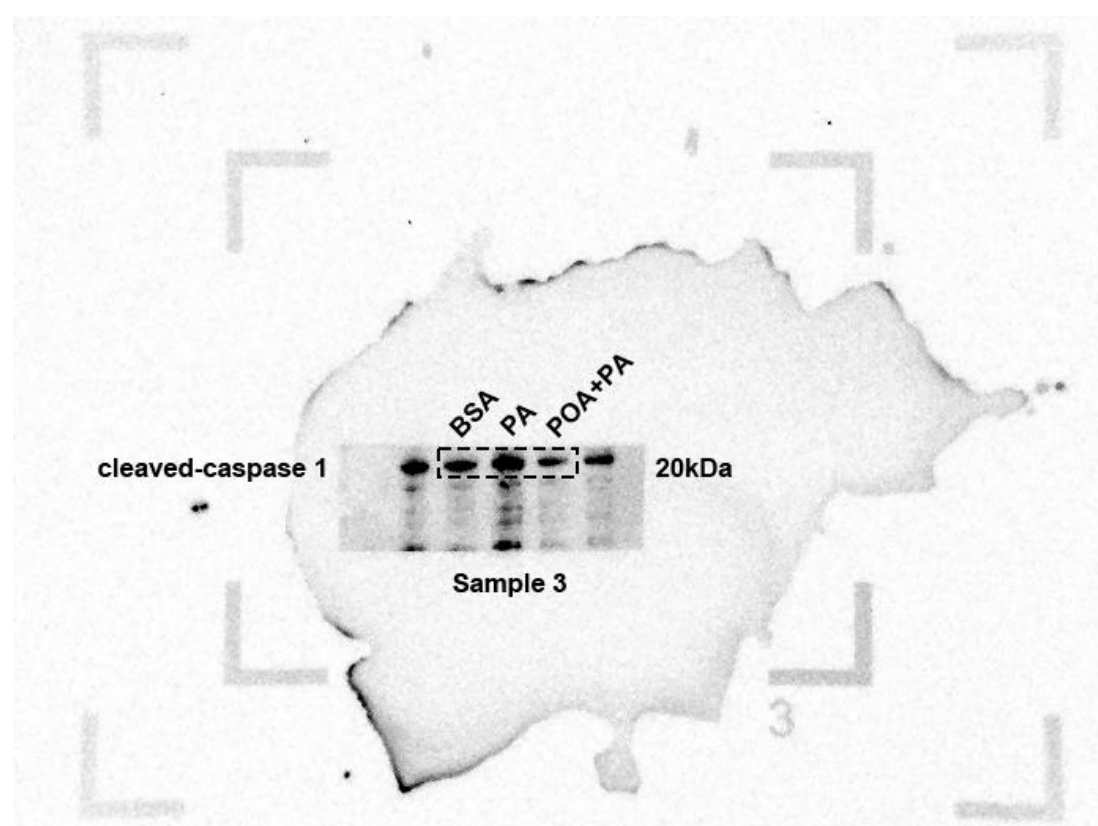

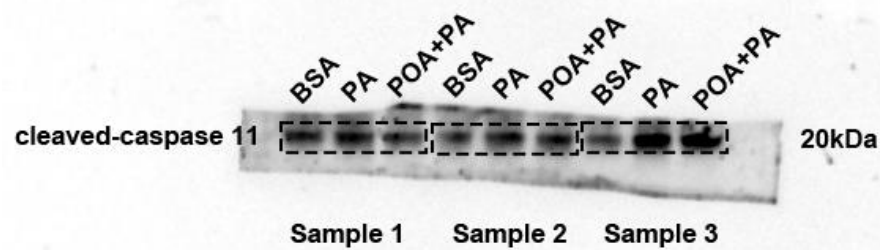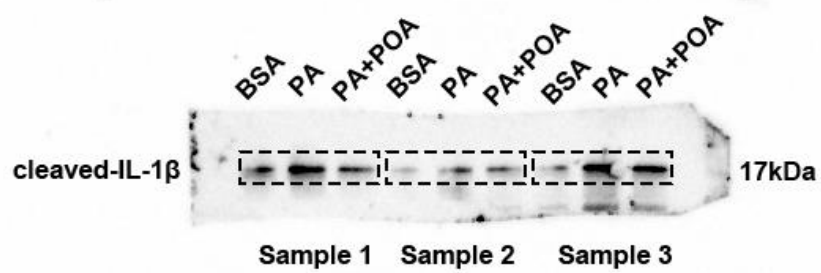

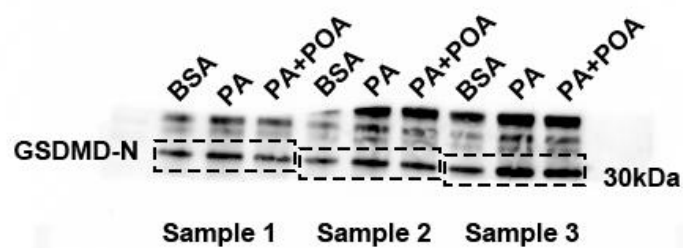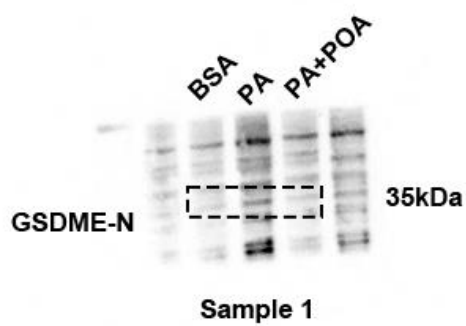

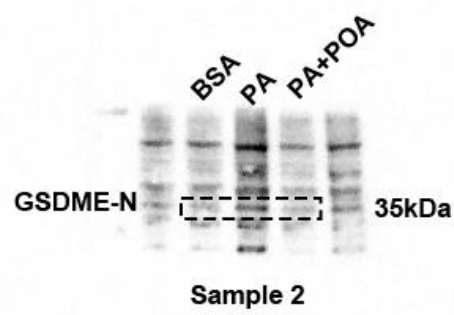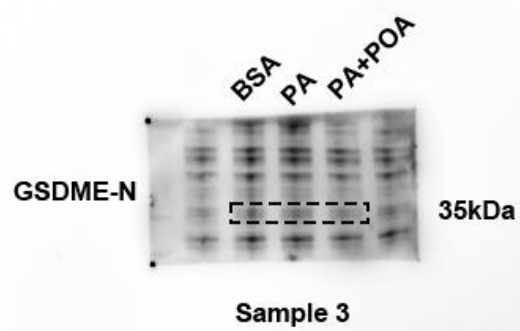

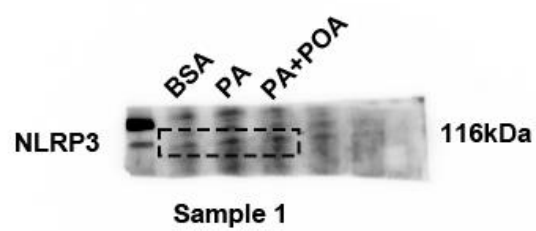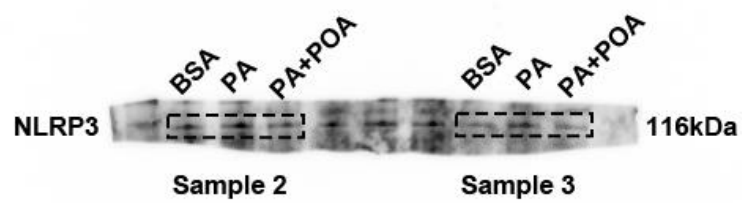

Supplement: S1 Raw images — (PDF) [file pone.0297031.s002.pdf]
